# Cystic Echinococcosis in Hospitalized Adult Patients from Western Romania: 2007–2022

**DOI:** 10.3390/microorganisms11102388

**Published:** 2023-09-25

**Authors:** Ana Alexandra Paduraru, Maria Alina Lupu, Laurentiu Sima, Gabriel Veniamin Cozma, Sorin Dan Olariu, Sorin Dan Chiriac, Bogdan Dan Totolici, Catalin Alexandru Pirvu, Fulger Lazar, Alexandru Nesiu, Alin Gabriel Mihu, Alin Adrian Cumpanas, Octavian Marius Cretu, Tudor Rares Olariu

**Affiliations:** 1 Discipline of Parasitology, Department of Infectious Diseases, Victor Babes University of Medicine and Pharmacy, 300041 Timisoara, Romanialupu.alina@umft.ro (M.A.L.); alin.mihu@umft.ro (A.G.M.); rolariu@umft.ro (T.R.O.); 2 Center for Diagnosis and Study of Parasitic Diseases, Department of Infectious Disease, Victor Babes University of Medicine and Pharmacy, 300041 Timisoara, Romania; 3 Patogen Preventia, 300124 Timisoara, Romania; 4 Clinical Laboratory, Municipal Clinical Emergency Hospital, 300254 Timisoara, Romania; 5 Clinical Laboratory, Institute of Cardiovascular Diseases, 300310 Timisoara, Romania; 6 Discipline of Surgical Semiology I and Thoracic Surgery, Department of Surgery I, Victor Babes University of Medicine and Pharmacy, 300041 Timisoara, Romaniagabriel.cozma@umft.ro (G.V.C.); 7 General Surgery Clinic, Municipal Clinical Emergency Hospital, 300254 Timisoara, Romania; 8 Thoracic Surgery Clinic, Municipal Clinical Emergency Hospital, 300254 Timisoara, Romania; 9 General Surgery Clinic, County Clinical Emergency Hospital, 300254 Timisoara, Romania; olariu.sorin@umft.ro (S.D.O.);; 10 Discipline of Surgery I, Department of Surgery II, Victor Babes University of Medicine and Pharmacy, 300041 Timisoara, Romania; 11 Discipline of Surgery III, Department of Surgery II, Victor Babes University of Medicine and Pharmacy, 300041 Timisoara, Romania; 12 Department of General Surgery, Vasile Goldis Western University of Medicine and Pharmacy, 310025 Arad, Romania; 13 Discipline of Surgical Emergencies, Department of Surgery II, Victor Babes University of Medicine and Pharmacy, 300041 Timisoara, Romania; 14 Discipline of Surgery II, Department of Surgery II, Victor Babes University of Medicine and Pharmacy, 300041 Timisoara, Romania; 15 Department of Biology and Life Sciences, Vasile Goldis Western University of Medicine and Pharmacy, 310025 Arad, Romania; alexnesiu@yahoo.com; 16 Department of Urology, Arad County Emergency Clinical Hospital, 310037 Arad, Romania; 17 Discipline of Urology, Department XV, Victor Babes University of Medicine and Pharmacy, 300041 Timisoara, Romania

**Keywords:** *Echinococcus granulosus*, parasitic disease, zoonosis, epidemiology, hydatid disease

## Abstract

Cystic echinococcosis (CE) is a neglected parasitic disease caused by the tapeworm *Echinococcus granulosus*. The aim of this study was to assess the epidemiological features of human cystic echinococcosis in patients from Western Romania. We retrospectively investigated the medical records of patients hospitalized with CE between 1 January 2007 and 1 September 2022. A total of 366 patients (range 18–90 years) were recorded. The number of hospitalized individuals was higher in patients aged 50–59 years (83/366, 22.7%), in females (194/366, 53%), and in residents of rural areas (225/366, 61.5%). The liver was the most common localization of the cysts (302/366, 82.5%). Ninety-eight patients (26.8%) presented complications, including biliary fistula, allergies, and infection of the cyst. Patients with complications had a longer mean hospital stay (15.7 ± 8.3 days) compared to patients without complications (11.5 ± 7.3 days) (*p* < 0.001). The results of this study revealed that patients diagnosed with CE required hospitalization and extended medical care, indicating that this zoonotic disease remains a significant public health problem in Western Romania. Public health authorities should enhance CE surveillance by implementing control programs and mandatory notification of new cases.

## 1. Introduction

Human echinococcosis is a neglected parasitic disease caused by the larval stages of *Echinococcus* tapeworms. Cystic echinococcosis (CE) is caused by the *Echinococcus granulosus* tapeworm [1,2,3]. The definitive hosts of *E. granulosus* are canids, infected through the ingestion of viscera containing viable hydatid cysts. Livestock animals (sheep, cattle, pigs, horses, and goats) are the intermediate hosts of the parasite and harbor the parasitic metacestode [2,3,4]. The interaction between predators and prey is essential to the life cycle of *E. granulosus* [1]. Humans are considered accidental hosts as they become infected by ingesting the parasitic eggs and are less likely to be involved in further transmission of disease [3,5]. Humans get the infection through consumption of food, water, or soil contaminated with dog feces containing parasitic eggs [6]. Moreover, eggs may remain attached to dogs’ fur when eliminated with feces. Close contact with dogs can lead to accidental ingestion of these eggs [7].

Data from the literature mention that pathogenesis processes linked with *Echinococcus* infections may be used to analyze the interactions between opposing forces generated by humoral and cellular immunostimulant and immunosuppressive mediators. It was observed that through the inhibitory action of Th2 and T reg immune responses, the host defenses may be avoided, permitting chronic worm infection [8]. Cystic echinococcosis is distinguished by the growth of single or multiple hydatid cysts [9]. Up to 80% of the cases are characterized by the development of solitary cysts in single organs [5]. Hydatid cysts remain asymptomatic for a long time due to their slow growth, and clinical manifestations depend on the organ involved [10,11]. They are usually diagnosed after months or even years [4]. Although any organ can be affected, hydatid cysts are mostly found in the liver (70%) and lungs (20%) [5]. Hepatomegaly, portal hypertension, cholestasis, biliary cirrhosis, ascites, and several other manifestations (upper abdominal pain, abdominal distention, loss of appetite, jaundice) have been reported in patients with hepatic cysts. Cysts rupture in the biliary tree, which may result in cholestasis and cholangitis, or into the peritoneum, resulting in anaphylaxis or secondary CE. Abscess may develop if cysts become infected. Symptoms of pulmonary cysts include chronic cough, expectoration, dyspnea, hemoptysis, pleuritis, and lung abscess [1,4].

Imaging techniques such as ultrasonography, radiology, computerized tomography, or magnetic resonance imaging are important tools in diagnosing cystic echinococcosis. Serology is based on finding the specific antibodies in the serum of a patient, and along with the imaging techniques help in establishing the diagnosis [3,4]. Still, specific anti-*Echinococcus* antibody production depends on various factors like the individual immune response, the affected organ, or the number of hydatid cysts [12].

Treatment of cystic echinococcosis depends on multiple factors, like localization and/or number of cysts, cystic developmental stage, size, the presence of complications, and patient’s clinical condition [3]. Until recently, surgery was considered the treatment of choice. Nowadays, the minimally invasive puncture-aspiration-injection-re-aspiration (PAIR) procedure, chemotherapy, and the “watch-and-wait” approach are also described as therapeutic options [3,10]. Surgery is efficient in most cases. However, it may not be an option in several circumstances, mainly when a patient has many cysts in various organs, when there is a high surgical risk, or when there are insufficient facilities to perform the intervention [4]. Surgical approaches that do not include total removal of the cyst should be supplemented by the use of protoscolicidal agents [4]. The PAIR technique is a minimally invasive approach that includes: i. percutaneous puncture of the hydatid cyst performed under ultrasound guidance; ii. partial fluid aspiration; iii. inoculation of a protoscoicidal agent; and iv. re-aspiration [13].

Chemotherapy using benzimidazoles is the chosen therapy option for some individuals with inoperable cysts, multiple cysts, or multiple organ involvement [4]. It appears to be more efficient in younger patients than in elderly ones [14]. Albendazole therapy is indicated for the PAIR technique or surgery, one week before and two months following the procedure [1]. The alternative drug for patients with important hepatic side effects is mebendazole [1]. 

Regardless of the improvements in surgical techniques and the use of chemotherapy, recurrence is still one of the main issues in the treatment of hydatid disease [15]. Recurrence rates in CE vary widely from 0% to 22% and are noticed at intervals of 3 months to 20 years after the initial surgery. Disease recurrence may be associated with severe complications such as intrabiliary rupture, anaphylaxis, and pyogenic infection [15].

Cystic echinococcosis has a worldwide distribution, is found mainly in rural and pastoral areas, and is associated with poor health education and living conditions [3,4]. According to the WHO Foodborne Disease Burden Epidemiology Reference Group (FERG), echinococcosis is responsible for 19 300 deaths and 871 000 disability-adjusted life-years (DALYs) worldwide annually [16]. CE not only causes severe illness and mortality in people, but it also causes economic losses. In humans, CE may have a series of negative effects, including direct financial expenses for diagnosis, hospitalization, surgical or percutaneous treatments, post-treatment care, as well as indirect costs for mortality, pain, loss of working days, social consequences of disability, and abandonment of agricultural activities by affected or at-risk individuals [17]. Livestock losses are due to organ seizures, decreased carcass weight, production of milk, fertility rates, and wool [18,19].

Chile, Uruguay, Argentina, southern Brazil, central Asia, western China, East Africa, and the Mediterranean region are considered endemic areas for cystic echinococcosis [3,5]. The annual incidence in endemic areas can reach 200 per 100,000 people [20]. The high endemicity in these areas is mostly associated with the large number of potential intermediate hosts, animal husbandry, home slaughtering, consumption of unwashed fruits or vegetables, the presence of stray dogs, and low knowledge of the disease [21]. 

Romania was also included by the World Health Organization [16] on the list of endemic countries [16,22,23]. The endemic occurrence of CE was previously reported in several Romanian regions [24,25]. An epidemiological research conducted between 1985 and 1997 in southwestern Romania revealed that the mean annual CE incidence was 5.1 cases/100,000 with a peak incidence of 9.5 cases/100,000 individuals in 1997 [26,27]. In Cluj County, northwestern Romania, the CE incidence varied between 3.9 and 9.5/100,000 during the period 1991- 2008 [28]. Between 2004 and 2010, a mean annual incidence of 3.3/100,000 was registered in two counties in southwestern Romania (Caras-Severin and Hunedoara) [26]. Of note, in Romania, 45.7% of the population lives in rural area, and approximately 30% of the population is involved in the agricultural sector [29]. According to Eurostat, Romania was in 2021 the second largest sheep breeder in the European Union, after Spain [30].

The aim of this study was to assess the epidemiological features of human cystic echinococcosis in patients hospitalized in surgical clinics in Western Romania for a period of 15 years.

## 2. Materials and Methods

### 2.1. Geographical Area of the Study

**Figure 1:**
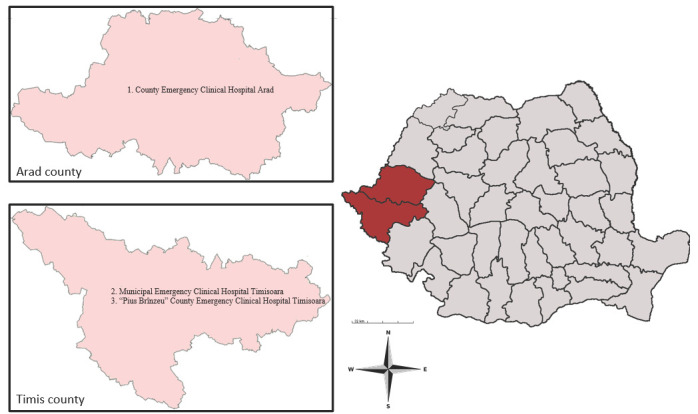
Map of Arad and Timis counties, showing the hospitals where the survey was conducted: (1) County Emergency Clinical Hospital Arad; (2) Municipal Emergency Clinical Hospital Timisoara; (3) “Pius Brînzeu” County Emergency Clinical Hospital Timisoara.

This study was performed in Arad County and Timis County. Both counties are situated in Western Romania and surrounded by four Romanian counties (Bihor, Alba, Hunedoara, and Caras-Severin) and districts from Hungary and Serbia, respectively [31,32]. These two Romanian counties cover an area of 16,450.7 km^2^, with a total population of 1,224,186 inhabitants. This geographic region has a temperate continental climate with Mediterranean and oceanic influences (Figure 1) (INS).

### 2.2. Data Collection

This retrospective study included adult patients aged ≥18 years diagnosed and hospitalized with cystic echinococcosis in Western Romania between 1 January 2007 and 1 September 2022. 

All medical records on cystic echinococcosis from three major teaching hospitals (County Emergency Clinical Hospital Arad, Municipal Emergency Clinical Hospital Timisoara, and “Pius Brînzeu” County Emergency Clinical Hospital Timisoara) referral centers for general and thoracic surgery in Western Romania were reviewed.

Data regarding age, gender, area of residence, length of hospital stay, number of hospitalizations due to CE, cyst localization, number of cysts, investigations performed, complications, and treatment were collected and evaluated. Patients were grouped as follows according to their age: 18–29 years, 30–39 years, 40–49 years, 50–59 years, ≥60 years. 

According to the medical records, the diagnosis was established based on imaging techniques (ultrasonography, radiology, computerized tomography) and confirmed by anatomopathological examination. Anti-*Echinococcus*-specific antibody detection using enzyme-linked immunosorbent assays (ELISA) was recorded in only 37/366 (10.1%) of the medical charts.

### 2.3. Statistical Analyses

All collected data were compiled in a Microsoft 365 Excel database, version 2205 (Microsoft Corp., Redmond, WA). Statistical analyses were performed using the software packages EpiInfo (v. 7.2, CDC, Atlanta, GA, USA, 2018) and MedCalc for Windows (v. 20.015, MedCalc Software, Ostend, Belgium). The chi-squared test, two-tailed Fisher’s exact test, or Mantel–Haenszel test, as appropriate, were used to evaluate associations between the studied groups, while Student’s t-test was used to assess differences between means in the studied groups.

Incidence rates of CE were calculated as the average annual number of cases per 100,000 inhabitants, according to the annual population number provided by the Romanian National Institute of Statistics (INS, 2023). To analyze trends in the number of cases and incidence of the disease over time, a linear regression analysis was performed. A *p*-value < 0.05 was considered statistically significant.

The study was conducted following the ethical principles of the Declaration of Helsinki and approved by the Ethics Committee of the Victor Babes University of Medicine and Pharmacy in Timisoara, Romania.

## 3. Results

A total of 366 hospitalized patients were recorded with cystic echinococcosis in Western Romania between 2007 and 2022. Patients included in the study were aged between 18 and 90 years (mean age 46.8 ± 16.2 years), with the age group 50–59 years being more frequently hospitalized (83/366, 22.7%). However, individuals < 50 years old accounted for 202/366 (55.2%) of the cases (Table 1). When comparing the mean ages of females (47.4 ± 16.8 years) and males (46.3 ± 15.7 years), no statistical difference was observed (*p* = 0.52).

The number of hospitalized cases was apparently higher in females (194/366,53%) and in patients residing in rural areas (225/366,61.5%). However, no significant difference was found between area of residence and gender (*p* = 0.48), between gender and age groups (*p* = 0.77), or between area of residence and age groups (*p* = 0.08) (Table 2).

**Figure 2:**
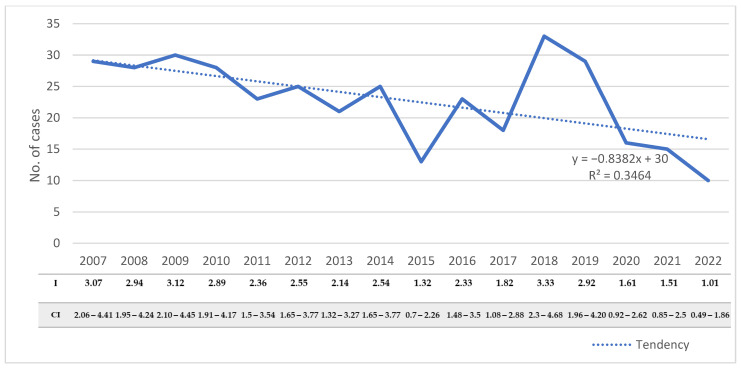
Distribution of CE cases with the corresponding incidences and confidence intervals in adults from Western Romania between 2007 and 2022. R^2^—goodness-of-fit measure for the linear regression equation; I—incidence; CI—95% confidence interval.

During the study period, the number of hospitalized cases varied from 10 to 33 cases/year, with an average annual number of 22.9 ± 6.8. The number of hospitalized cases decreased significantly during the studied period, from 29 cases in 2007 to 10 cases in 2022 (R^2^ = 0.346, *p* = 0.02) (Figure 2). The decreasing trend was also observed when we assessed the incidence rates of the disease in the adult population (R^2^ = 0.389, *p* = 0.009).

The length of hospital stay varied between 1 and 47 days, with a mean of 12.6 ± 7.8 days. A total of 91 (24.8%) of the 366 patients were hospitalized between 1 and 7 days, 158 (43.2%) were hospitalized between 8 and 14 days, and 117 (32%) were hospitalized for more than 14 days. We observed an association between the affected organ and the hospitalization period (ꭕ^2^ = 31.74, *p* < 0.001). Thirty of the 48 (62.5%) patients with pulmonary cysts had more than 14 days of hospitalization compared to patients with liver (82/302, 27.2%), kidney (2/4, 50%), or spleen (2/6, 33.3%) cysts, with a similar length of hospital stay.

The liver was the most affected organ (302/366, 82.5%), followed by the lungs (48/366, 13.1%), spleen (6/366, 1.6%), kidneys (4/366, 1.1%), and other anatomical sites, including the retroperitoneum, peritoneum, muscle, and bone, in 6/366 (1.6%) of the cases. In CE with primary liver involvement, the right lobe was affected in 219/302 (72.5%) cases, the left lobe in 57/302 (18.9%), and both lobes in 26/302 (8.6%) cases. In primary lung involvement, the left lung was affected in 24/48 (50%) patients, the right lung in 21/48 (43.8%), and both lungs in 3/48 (6.2%). We observed a significant statistical association between gender and the affected organ (ꭕ^2^ = 12.8, *p* = 0.01). Of the patients with liver cysts, females accounted for 171/302 (56.6%) of the cases, while of those with lung and kidney involvement, hydatic cysts were more frequently reported in males, in 29/48 (60.4%) and 4/4 (100%), respectively.

Multiple organ involvement was noted in 23/366 (6.3%) patients. The most frequent associations were liver-lung and liver-peritoneum in 6/23 (26.1%) of the cases, respectively. A triple association of liver-lung-spleen and liver-kidney-spleen was noticed in 1/23 (4.3%) cases, respectively (Table 3).

Complications were reported in 98/366 (26.8%) cases. The main reported complications were biliary fistula (45/98, 45.9%), allergies (15/98, 15.3%), and cyst infection (11/98, 11.2%). There was a statistically significant association between the presence of complications and the length of hospital stay (ꭕ^2^ = 24.16, *p* < 0.001). Fifty of the 98 (51%) patients with complications had over 14 days of hospitalization, while only 67/268 (25%) patients without complications had over 14 days of hospitalization (*p* < 0.001). Moreover, patients with complications had a longer mean hospital stay (15.7 ± 8.3 days) compared to patients without complications (11.5 ± 7.3 days) (*p* < 0.001).

In all cases, the diagnosis of CE was based on imaging techniques such as ultrasonography, radiography, and computerized tomography and confirmed by demonstrating the presence of protoscoleces in post-surgery samples. Twenty-eight of the 37 (75.7%) serologically tested patients had anti- *Echinococcus* antibodies.

Surgery was the treatment of choice in 298/366 (81.4%) cases. The PAIR technique was chosen in 53 (14.5%) cases, Albendazole therapy alone was used in 8 (2.2%) cases, and in 7 (1.9%) cases, the ”Watch-and-Wait” approach was preferred (Table 4). Chemotherapy with Albendazole as adjuvant therapy was used in 99/366 (27%) cases (Table 4). In patients with liver cysts, Lagrot partial pericystectomy was performed in 180/302 (59.6%), total cystectomy in 42/302 (13.9%), PAIR technique in 53/302 (17.5%), segmentectomy in 12/302 (4%), oral therapy in 8/302 (2.6%), and the “Watch-and-Wait” approach in 7/302 (2.3%). In patients with lung involvement, cystectomy was performed in 40/48 (83.4%) cases, partial pericystectomy in 4/48 (8.3%), and segmentectomy in 4/48 (8.3%). In spleen involvement, 4 (66.7%) of 6 patients underwent splenectomy and 2/6 (33.3%) underwent partial pericystectomy.

A total of 83 of the 366 (22.7%) patients experienced recurrences. A total of 63 of the 83 (75.9%) patients had two hospitalizations, while the remaining patients (24.1%) had more than two hospital admissions. In patients who received Albendazole therapy, recurrence was identified in 30/366 (8.2%) cases. However, no statistically significant difference was observed between patients who were administered drug therapy compared to those who did not receive any Albendazole therapy regarding the presence of recurrences (*p* = 0.12).

## 4. Discussion

According to the World Health Organization, human cystic echinococcosis is one of the most important neglected zoonoses and represents a serious socio-economic problem in many parts of the world [2]. Important morbidity and mortality due to CE were reported, especially in developing countries [33]. Human echinococcosis was reported in most European countries except Denmark, Iceland, and Ireland [34]. The highest rates in Europe were registered in the Mediterranean region [34]. High rates were also reported in Bulgaria, where the average annual incidence reached up to 6.7/100,000 inhabitants [34,35]. Recently, in Eastern European countries, a lower incidence of CE has been reported. For instance, in Eastern Hungary, Dezsényi and colleagues [3] found an incidence of 1.49 cases/100,000 inhabitants, while in Serbia, another country with CE-endemic regions, the incidence was estimated at 0.73/100,000 [12].

Romania is considered one of the endemic countries for cystic echinococcosis, with an incidence rate that reached 5.6 cases/100,000 inhabitants [27]. In Romania, the transmission of CE was associated with various variables like a temperate climate, a high percentage of people working in the agricultural sector, unsupervised home slaughtering of livestock (particularly sheep and pigs), the presence of a significant number of feral dogs, or the absence of antiparasitic treatment in dogs. [23,36,37,38].

In Romania, CE was reported in animals such as sheep, swine, and cattle. In a study performed between 1998 and 2003, field data obtained from Timis District slaughterhouses revealed prevalences of 22.36% in cattle, 5.83% in sheep, and 4.32% in swine [39]. Between 2009 and 2011, in northeastern and southern Romania, Mitrea and colleagues [23] reported prevalences of 49.87% and 32.34% in sheep and cattle, respectively. In a recent study performed between 2020 and 2021, Darabus and colleagues found a CE prevalence of 2.45% in cattle [40]. This reduction in prevalence may indicate an improvement in Romania’s sanitary-veterinary control efforts at the farm level. In addition, advances in canine population control programs (e.g., microchipping of dogs) have increased the possibility for veterinarians to instruct owners to deworm their dogs [40]. Noteworthy, the occurrence of human CE in a geographical region has been linked to the level of sheep-raising activities, especially the density of sheep in the region; this has also been linked to the rates of human CE-related hospital admissions [41].

Hospital-admitted CE cases are recorded in the medical databases, but the precise rate of infection is unknown since many people avoid seeking medical attention due to financial reasons. As a result, many cases are likely to go undiagnosed and unreported, increasing morbidity and death due to CE [42]. In Romania, there are no official national programs for the control of *E. granulosus,* and there is no obligation to report the new cases. Therefore, hospital discharge records are the only data source available for detecting CE cases in humans. Ultrasound-based screening programs may provide a more precise picture of the epidemiology of CE at the population level. Such investigations permit the identification of ongoing transmission of the parasite and can offer accurate illness prevalence estimations [41,43].

In this study, more than half of the hospitalized patients were aged <50 years. Interesting, about 20% of the patients aged 18–29 years, indicating that subjects in this age group acquired the infection early in childhood. Similarly, in Argentina and Pakistan, CE cases were more commonly reported in young adults (15–29 years in Argentina, 21–30 years in Pakistan) [2,42,44]. A higher susceptibility to CE at young ages might be associated with early exposure to the parasite from contact with dogs, playing in contaminated soil, or involvement in animal grazing [42,44]. Assessment of hospitalized cases according to age groups revealed that almost half of the patients with CE in our study were over 50 years old. An explanation may be that the disease develops over a long period of time, implying a lag between infection and diagnosis [45]. However, the absence of an evident trend in the distribution of CE between the different age groups suggests that CE can manifest at any age [26,42,46,47].

The results of the present study showed a decreasing trend in the number of cases and also in the incidence of the disease between 2007 and 2022. This could be partially attributed to higher awareness of illness in the local community and among healthcare professionals [48]. Another explanation for the decreasing trend could be the implementation of stronger standards concerning safety, food, and health since the accession of Romania to the European Union (EU) in 2007 [49]. Former investigators reported similar results to our findings. Petropoulos and colleagues [50] identified a downward trend in the number of cases during a 39-year period of time in Greece, while Mustapayeva and colleagues [48] observed a decreasing trendline in the incidence of surgical CE cases in Kazakhstan between 2007 and 2016. However, it is important to mention that the significant decrease in cases between 2020 and 2022 in our study might be impacted by the COVID-19 pandemic when hospital admissions were limited.

Regarding the gender distribution of CE, more than half of our patients were female. Similar results were also reported by other authors [12,42,44,51,52,53]. Moreover, in the cross-sectional study on healthy blood donors from Western Romania, females tended to have a higher seropositivity rate compared to males [54]. The high exposure to the parasite in females may be explained by domestic activities such as feeding and close contact with dogs, cleaning dog feces, or contact with contaminated soil [42,44].

Our findings are in concordance with most studies regarding the prevalence of the infection in rural areas. Studies conducted in Palestine, Iran, or Serbia have shown a higher rate of infection in people residing in rural areas [52,53,55]. The distribution of cystic echinococcosis is usually associated with rural areas. Poor rural communities are susceptible to infection due to little knowledge of the disease [34,56]. Farming, especially raising sheep, and home slaughtering are considered important risk factors in developing CE [34,56]. Moreover, a crucial role in the maintenance of the *Echinococcus* life cycle is played by dogs with little veterinary attention found next to the livestock [51,56]. The *E. granulosus* life cycle in stray dogs and sheep is maintained by home slaughtering of animals, feeding uncooked viscera to stray dogs near abattoirs, lack of abattoir disposal pits, and low public knowledge on the disease. All these factors contribute to potential environmental contamination [42].

Most of the patients in our study group were hospitalized between 8 and 14 days, and the mean length of hospital stay was 12.6 ± 7.8 days. However, it is important to note that almost a third of the patients had a hospital stay longer than 14 days. In Italy, during 2001–2012, 41% of the patients with CE were hospitalized for 1–7 days, and the mean length of hospitalization was 12.1 ± 12.1 [57]. In Turkey, Akkucuk and colleagues [58] identified a shorter mean length of hospitalization (5.42 ± 3.16 days) in patients with liver hydatid disease.

In the present study, single-organ involvement was observed in the vast majority of the patients. Brundu and colleagues [57] reported similar results, with 88.6% of the cases having cysts in a single organ. Our data indicate that cystic lesions occurred most frequently in the liver (302/366, 82.5%), especially the right lobe (219/302, 72.5%). This outcome was similar to previous findings documented by other authors [59,60]. Frequent hepatic localization might be attributed to the fact that *Echinococcus* oncospheres must pass first via the liver capillaries and usually remain trapped in the tissue and develop into cysts [56]. Moreover, the right hepatic lobe may be more affected due to portal blood flow [61]. Regarding lung localization, in our study group, hydatic cysts in the lungs were noted in 13% of 366 cases. Previous studies have shown that the right lung may be affected in most cases of pulmonary CE [62]. Interestingly, in our study group, the left lung was the most frequently reported localization compared to the right lung.

The diagnosis of CE is usually based on imaging techniques. Serology may help in supporting the diagnosis, or it may be an indicator of recurrence or relapse [63]. It is known that serology has lower sensitivity than imaging techniques [64]. However, serological studies are performed worldwide to assess the prevalence of infection or to help establish the diagnosis [65,66]. Antibody production mostly depends on the organ affected, the number of cysts, and the presence of complications [63,67]. Serological surveys showed that 10–20% of patients with hepatic involvement and approximately 40% with pulmonary involvement do not develop detectable specific antibodies [4]. In this study, anti-*Echinococcus* antibodies were positive in 75% of the tested patients. Similar results were reported in Hungary, where anti-*Echinococcus* antibodies were detected in 17 of the 27 (62.96%) tested patients [3], and in Spain, where 48 of the 93 (51.6%) investigated patients were seropositive [68].

Our findings revealed that more than a quarter of the patients had complications. Recently, in a study conducted in Spain, the reported complication rate was 27/151 (34.6%) [68]. Usually, cysts are diagnosed by incidental radiological or clinical examination [69]. The classification of complications according to their pathogenesis is: mechanical, caused by compression or rupture of the cyst; immunological, caused by allergic reactions to the cyst; and superinfection due to the presence of microorganisms [70]. Intrabiliary rupture of the cyst was found to be the most frequent complication of CE, accounting for incidence rates between 3 and 17% [69]. Rupture usually occurs in the right hepatic duct. Communication often occurs through small fissures; therefore, it is difficult to tell the exact location of the fissure [69]. Bacterial infection is usually a rare complication due to the pericyst avascularity and the lack of communication between the host vascular system and the endocyst. A key requirement for bacterial infection is the rupture of the endocyst and pericyst [69]. Escolà-Vergé and colleagues [68] reported cyst infection as the most common complication in patients hospitalized for CE in Spain. In our study, biliary fistula was the most frequent complication, being described in 45/98 (45.9%) of the patients.

The clinical data recorded in the present study indicate that in Western Romania, surgery was the main option in the management of CE cases. Surgery, using various techniques, has the capacity to remove the cysts, resulting in a full recovery [4]. Albendazole was used in 107 (29.2%) of the 366 patients: main therapy in 8 (2.2%) and adjuvant therapy in 99 (27%). Albendazole is usually used to decrease the risk of relapse, especially in patients undergoing invasive surgical treatment [15,57]. However, 83 (22.7%) of the patients in our study reported having recurrences. Brundu and colleagues [57] also identified high rates of recurrences in a retrospective study conducted in Italy, with 24% of the patients having two hospital stays and 5% having three or more hospitalizations. Lower rates of relapse were reported by Amado-Diago and colleagues [71] and Velasco-Tirado and colleagues [15] in Spain, who identified recurrences in 10.5% and 11.5% cases, respectively. In addition to the low use of Albendazole post-surgery, another important cause for recurrences may be the spillage of hydatid cyst fluid in the abdominal cavity during surgery [72].

Some limitations of this study should be considered. First, there is the retrospective nature of the study. Our results are supported by the available data collected from patients’ medical records. Information regarding other epidemiological aspects that could be potential risk factors for the infection was not included in all the charts. Second, the database included only hospitalized cases. Therefore, the number of our patients does not reflect the total number of CE cases in the two counties, which is probably higher, as a series of cases remain asymptomatic and might go undiagnosed [54].

## 5. Conclusions

The current retrospective study brings new and important data regarding the epidemiology of CE in Western Romania. Patients diagnosed with CE required hospitalization and extended medical care, indicating that this zoonotic disease remains a significant public health problem in this region.

In reference hospitals, multidisciplinary teams should form to improve the diagnosis and management of CE cases. The implementation of a surveillance system with mandatory notification of new cases should help in monitoring the trends of the disease and improve the patients’ follow-up. Further epidemiological investigation of CE-confirmed cases with a focus on molecular particularities is highly recommended to better understand the transmission patterns of the parasite in Romania. 

Furthermore, effective communication between human doctors and veterinarians is essential to improving knowledge on the disease and its prevention measures, especially among at-risk populations. Public health strategies for human and animal infection should be optimized, including control programs and increasing awareness regarding the transmission of the parasite.

## Figures and Tables

**Table 1:** Distribution of CE cases in Western Romania according to age groups and gender (*n* = 366).

Variable	Gender		Total (%)
	Male (%)	Female (%)	
18–29	30 (42.9)	40 (57.1)	70 (19.1)
30–39	31 (51.7)	29 (48.3)	60 (16.4)
40–49	37 (51.4)	35 (48.6)	72 (19.7)
50–59	37 (44.6)	46 (55.4)	83 (22.7)
≥60	37 (45.7)	44 (54.3)	81 (22.1)
**Total**	172 (47)	194 (53)	366 (100)

**Table 2:** Distribution of CE cases in Western Romania according to gender and area of residence (*n* = 366).

Variable	Area of Residence		Total (%)
	Rural (%)	Urban (%)	
**Gender**			
Female	116 (59.8)	78 (40.2)	194 (53)
Male	109 (63.4)	63 (36.6)	172 (47)
**Total (%)**	225 (61.5)	141 (38.5)	366 (100)

**Table 3:** Distribution of cystic echinococcosis cases by anatomical localization of the cysts.

Localization of the Cyst	No. of Cases *n* = 366 (%)
Liver	302 (82.5)
Lungs	48 (13.1)
Spleen	6 (1.6)
Kidneys	4 (1.1)
Other *	6 (1.6)
**Multiple organ involvement**	**No. of cases *n* = 23 (%)**
Liver associated with:	
Lung	6 (26.1)
Peritoneum	6 (26.1)
Spleen	2 (8.7)
Pelvis	2 (8.7)
Iliopsoas muscle	1 (4.3)
Gallbladder	1 (4.3)
Kidney	1 (4.3)
Kidney and spleen	1 (4.3)
Lung and spleen	1 (4.3)
Lung associated with heart	1 (4.3)
Spleen associated with retroperitoneum	1 (4.3)

* Other: peritoneum (3 cases), retroperitoneum (1 case), muscle (1 case), and bone (1 case).

**Table 4:** Distribution of CE cases according to treatment instituted (*n* = 366).

Variable	No. of Cases (%)
**Treatment**	
Surgery	298 (81.4)
PAIR	53 (14.5)
Only chemotherapy	8 (2.2)
Watch and wait	7 (1.9)
**Adjuvant therapy**	
Albendazole	99 (27)

## Data Availability

Data are available upon request.
